# Is there a correlation between follicle size and gene expression in cumulus cells and is gene expression an indicator of embryo development?

**DOI:** 10.1186/s12958-018-0388-0

**Published:** 2018-07-21

**Authors:** Semra Kahraman, Caroline Pirkevi Çetinkaya, Murat Çetinkaya, Mehmet Ali Tüfekçi, Cumhur Gökhan Ekmekçi, Markus Montag

**Affiliations:** 1 Istanbul Memorial Hospital, Assisted Reproductive Technologies and Reproductive Genetics Centre, Piyale Pasa Bulvari 34385 Okmeydani Sisli, Istanbul, Turkey; 2 ilabcomm GmbH, Eisenachstr. 34, 53757 Sankt Augustin, Germany

**Keywords:** Gene expression, Cumulus cells, Follicular size, Blastocyst

## Abstract

**Background:**

In an article published in 2017*,* we discussed the results of the first part of our study into the morphokinetic development of embryos in relation to follicle diameter and homogeneity of follicular development. Our findings showed that embryos coming from small follicles in heterogeneous cycles had significantly higher rates of arrest or failure to reach blastocyst than embryos coming from large follicles in homogenous cycles. The aim of this further study was to investigate the relationship between follicular size and gene expression of cumulus cells (CCs) and evaluate whether gene expression could be an indicator of embryo development.

**Methods:**

This study was based on 2495 COCs from 184 patients. CC expressions of five genes (*TNFAIP6*, *PTGS2*, *HAS2*, *PTX3* and *GDF9*) were studied by generalized linear mixed models (GLMMs) regarding follicular size. CC expressions were then separately analysed regarding patient-specific variables (age, BMI, AMH and follicular size) in relation to embryos reaching blastocyst (eRB) or top or good quality blastocysts (TQ + GQ) using GLMMs with logit link.

**Results:**

Follicular size significantly correlated with the potential of an oocyte to develop into a blastocyst: oocytes developing from large follicles were more than twice as likely to develop into an eRB than oocytes from small follicles (*p* < 0.001). Gene expression of *HAS2* and *GDF9* correlated with blastocyst quality when separately evaluated with follicular size and the patient specific variables of age, BMI and AMH. However, no such correlation was found in other gene expressions studied.

**Conclusions:**

Our findings suggest that differences in the expression of genes studied could be related to follicular size rather than to embryo quality. Although gene expression of *HAS2* and *GDF9* correlated with blastocyst quality, the only variable correlating with eRB and TQ and GQ blastocysts for each of these five models was follicular size.

**Trial registration:**

This prospective cohort study was registered at clinicaltrials.gov (NCT02230449).

**Electronic supplementary material:**

The online version of this article (10.1186/s12958-018-0388-0) contains supplementary material, which is available to authorized users.

## Background

We conducted a project between July 2014 and September 2015 investigating the relationship between the follicular size and oocyte competence using the morphokinetic and gene expression analyses of the same patient group.

In an article published in 2017*,* we discussed the results of the first part of our study into the morphokinetic development of embryos in relation to follicle diameter and homogeneity of follicular development [1]. Our findings showed that embryos coming from small follicles in heterogeneous cycles had significantly higher rates of arrest or failure to reach blastocyst than embryos coming from large follicles in homogenous cycles. Subsequently, we conducted this further study to compare gene expression between cumulus cells (CCs) obtained from follicles of different sizes, evaluating RNA concentrations of five well described genes involved in cumulus expansion/ ovulation (*HAS2*, *PTGS2*, *PTX3*, *TNFAIP6* and *GDF9*) [2–13].

There is increasing evidence that CCs play a crucial role in folliculogenesis and oocyte developmental competence acquisition [14–18]. Therefore, many studies correlating cumulus gene expression with different outcome measures such as oocyte maturity, fertilization, embryo development, implantation and pregnancy have been performed [2, 3, 16–24]. However, few investigate the relationship between gene expression and follicular size and embryo development to the blastocyst stage [25–27]. Recently, Nivet and colleagues [24], hypothesized that the combination of a follicle’s size and transcriptomic signature provide a reliable method to predict embryonic development. They concluded that follicular size had an impact on oocyte quality as measured by embryonic development and demonstrated that medium follicles yielded a better percentage of transferable embryos than large or small follicles.

The CC-derived candidate genes selected for the study were those which have been identified in previous studies as regulated by oocyte signals and as having important functions in cumulus expansion and metabolism [28–30].

These were: hyaluronan synthase 2 (*HAS2*), prostaglandin-endoperoxide synthase 2 (*PTGS2*), pentraxin-related protein 3 (*PTX3*), tumour necrosis factor alpha induced protein 6 (*TNFAIP6*) and growth differentiation factor 9 (*GDF9*).

*HAS2* is critical for the formation and expansion of the CC mass and expression levels correlate with early embryological development [2, 4]. Furthermore, *HAS2* is one of the four genes contained in the ranking method evaluated by Ekart and colleagues [19] for identifying good quality metaphase II (MII) oocytes that will ultimately provide a significantly better outcome regarding good blastocyst development and live birth compared with random selection. The measurement of *HAS2* and *GREM1* levels in CCs would reliably complement the morphological evaluation providing a useful tool for selecting oocytes with greater chances to be fertilized and develop in vitro. Furthermore, *HAS2* is considered to be a human pregnancy or live birth candidate biomarker with conflicting differential mRNA expression by qRT-PCR [2, 19, 31, 32].

In bovine and mice, *PTGS2* has a role in promoting cumulus expansion and oocyte competence [5–8]. In a landmark study comprising eight patients, *PTGS2*, like *HAS2*, was found to correlate with the morphology of human day-3 embryos [2]. The median expression for *PTGS2* was found to be threefold higher in CCs of oocytes that resulted in pregnancy [31]. Moreover, *PTGS2* expression was found to be significantly higher in CCs from oocytes that achieved term birth compared with those that failed to result in a successful pregnancy [31]. Previous studies have suggested that *PTGS2* expression in bovine and primate COCs may participate in the timing of maturation [7, 33] and oocyte quality [6]. Other studies have found an association between *PTGS2* and embryo morphological grade [2]. In mice, *Ptgs2* and the resultant prostaglandin E2 facilitate cumulus expansion through induction of cumulus matrix genes [5] and are important in the survival of mouse cumulus cells [8].

The *PTX3* gene product is another extracellular matrix protein that interacts with hyaluronan in the expanded cumulus matrix [34–36]. *PTX3* gene expression in CCs has been associated with oocyte/embryo competence and was determined as a potentially reliable predictor of embryo developmental competence [3]. Elevated *PTX3* expression has been found in CCs from oocytes that developed into normal appearing embryos on day 3 compared with those from oocytes that failed to fertilize in 16 patients [3]. However, another study failed to establish a correlation between *PTX3* expression and embryos with good or poor morphology [21]. Nonetheless, in other studies, expression of *PTX3* tended toward an association with pregnancy outcome [31, 37].

*TNFAIP6* is synthesized by cumulus and granulosa cells in the preovulatory follicle [9, 10]. It was selected because it is a *GDF9*-induced gene in periovulatory granulosa cells although its expression does not differ as a function of embryo quality and oocyte competence [2, 38, 39].

*GDF9* is an oocyte-secreted factor playing a key role in the process of follicular development from the recruitment of the primordial follicle to ovulation and even in corpus luteum formation [11–13]. It has been confirmed by many studies that *GDF9* is expressed both in oocytes and cumulus granulosa cells [40–42]. Increased levels of *GDF9* and *BMP15* expression have both been associated with positive pregnancy as well as with mature oocytes, fertilization rate and embryo quality [43].

The aim of our study was to investigate the relationship between follicular size and gene expression of CCs assessing these five candidate genes and to evaluate whether gene expression is an indicator of embryo development.

## Methods

### Patients

This prospective cohort study was registered at clinicaltrials.gov (NCT02230449) and received funding from the Grant for Fertility Innovation (GFI) in July 2014. It was conducted in a private IVF clinic between July 2014 and September 2015 and the analysis was based on 2495 COCs belonging to 184 patients, with culture until day 5. Our previous study on follicular size and morphokinetics analysed 2526 oocytes [1]. However, in this study three patients and their 31 oocytes were excluded from the expression analysis because their CC samples did not give interpretable results due to technical issues. The patients presented various infertility causes, all protocols were approved by the institutional review board and all patients gave their informed consent prior to their inclusion in the study. Patients were selected with inclusion criteria as similar as possible to the first part of the study [1]. Furthermore, the inclusion criteria specified good prognosis patients with at least 8 COCs in order to provide us with a sufficient number of blastocysts for statistical analyses.

Patients were selected based on inclusion criteria (age ≤ 39 years, body mass index (BMI) < 30 kg/m^2^, ≥ 8 COCs retrieved, < 2 previous treatment cycles, hCG trigger) and exclusion criteria (recurrent pregnancy loss, severe endometriosis, PGD or PGS, COC > 24, embryo transfer before day 5, PCOS, uterine anomaly, very severe sperm morphological abnormality such as dominantly macrocephal or globozoospermic sample or cryptozoospermia, ≤ 1million motile sperm cells in total ejaculate). In order to minimize bias, PCOS patients with an abnormally high number of COCs were excluded [44]. Patient characteristics are shown in Additional file 1: Table S1.

Female ages ranged between 18 and 38, with an average of 30.81 ± 4.26 years. AMH measurements ranged from 0.17 to 8.6 with a mean of 3.15 ± 1.80. BMI measurements ranged from 16.3 to 29.7 kg/m^2^ with a mean of 23.68 ± 3.00 kg/m^2^. This cycle was the first for 66.3% of the patients (*n* = 122) and the second for 33.7% of the patients (*n* = 62). Biochemical pregnancy was observed in 66.3% of the patients (*n* = 122), 53.3% (*n* = 98) had an ongoing pregnancy and finally, the live birth rate was 51.1% (*n* = 94).

### Ovarian stimulation

Ovarian stimulation was performed as described previously [1]. Briefly, recombinant FSH (rFSH; Gonal-F®; Merck Serono, Switzerland) was used at a dosage of 150 to 225 IU, depending on clinical and hormonal evaluations. A daily administration of 0.25 mg GnRH antagonist (Cetrotide®; Merck Serono, Switzerland) was started when at least one follicle reached 12–13 mm in size. Then, 250 μg recombinant hCG (Ovitrelle®; Merck Serono, Switzerland) was administered to achieve final oocyte maturation when at least three follicles reached a minimum mean diameter of 17 mm. Transvaginal ultrasound-guided oocyte retrieval was scheduled for 36 h later.

### Follicular size

Follicular size was defined as large, ≥17 mm and small, < 17 mm at the time of oocyte pick-up (OPU), as a pilot study of more than 2300 oocytes previously performed in our ART centre in 2012 suggested that 17 mm was the size where statistically significant differences were observed. This preliminary study showed a relative increase of 80% for the good and top-quality blastocyst rate (TQ + GQ) of oocytes deriving from large follicles when compared to small follicles if the cut-off was set as 17 mm. This difference disappeared when the cut-off was taken as 20 mm. Each follicular aspiration was performed by the same doctor to reduce any possible inaccuracy of measurement to a minimum.

### Cumulus oocyte complex (COC) collection and embryology

Oocyte retrieval, denudation, ICSI and embryo culture were performed as described previously [1]. In summary, on the day of OPU, follicles were aspirated individually. The gynaecologist who performed the pick-up notified the embryologist regarding the size of the follicle for each punctate. A second embryologist assisted the procedure to document the process of isolation, identification and positioning of COCs in the culture dish. Denudation was carried out by mechanical pipetting in ICSI cumulase® (Origio, Måløv, Denmark). Each COC was denuded separately and the maturation status was determined after denudation. Oocytes coming from large and small follicles were incubated separately in different dishes in the same incubator [1]. ICSI was performed at 400× magnification using Olympus IX70 and Olympus IX71 inverted microscopes. Embryo culture was carried out in a single step culture medium (Life Global®, Brussels, Belgium), supplemented with 10% plasmanate (Life Global®, Brussels, Belgium) in a time-lapse incubator (EmbryoScope™). Blastocysts were scored according to Gardner’s classification (114–120 h post-ICSI) and selected for transfer based on the final morphology and the score obtained from the morphokinetic ratios published by Çetinkaya and colleagues [45].

### Embryo transfer and outcomes of treatment cycles

After embryo transfer, for luteal phase support, patients received a twice daily dose of progesterone gel to be administered intravaginally (Crinone® 8%; Merck Serono, Switzerland). When pregnancy occurred, a daily dose was continued until the 10th week of gestation. Fourteen days after pick-up, serum β-hCG was measured. At 7 weeks, a transvaginal ultrasound was performed to monitor early pregnancy. The implantation rate was calculated by dividing the number of implanted embryos by the total number of transferred embryos. However, for analyses purposes, patients with two transferred embryos but resulting with only one sac were excluded from the analysis as we could not know which embryo had implanted and therefore would not have been able to analyse any possible correlation between follicular size, gene expression and embryo development. Implantation analysis was performed on transferred embryos where the number of gestational sacs matched the number of transferred embryos and on embryos where no biochemical pregnancy was achieved.

### Total RNA isolation from cumulus cells and reverse transcription

The CCs of each oocytes were separately numbered, frozen, and cryostored at − 20 °C in Cryo.S ™ Freezing Tubes (Greiner Bio-One, Germany) containing 150 μL of RNA *later* Stabilization Reagent (Qiagen, Germany). CCs were first incubated overnight in the reagent at 2–8 °C, then transferred to − 20 °C for storage until RNA extraction. Total RNA extraction of individual CCs was carried out using the Total RNA Purification kit (NORGEN, BIOTEK CORP., Canada) as recommended by the manufacturer and final elution of the total RNA was performed using 32 μl of RNase free water. Total RNA was quantified using a NanoDrop 2000c UV-Vis spectrophotometer (ThermoScientific, USA). The mean quantity of RNA per CC sample was 102.5 ± 44.3 ng. RNA from each sample was used to generate cDNA using the Transcriptor High Fidelity cDNA Synthesis Kit (Roche Diagnostics GmbH, Mannheim, Germany) with random hexamers, following the manufacturer’s instructions. Total RNA and/ or cDNA samples were stored at − 80 °C until use.

### Real-time PCR

Real-time PCR amplification reactions were carried out using a LightCycler 480 (Roche Diagnostics GmbH, Mannheim, Germany) with the LightCycler FastStart DNA Master SYBR Green I Kits (Roche Diagnostics GmbH, Mannheim, Germany). Real-time PCR was used to quantify the mRNA transcripts levels of *HAS2*, *PTGS2*, *PTX3*, *TNFAIP6* and *GDF9*. Oligonucleotide primer sequences are listed in Additional file 1: Table S2. Primers were designed to cross an exon-exon junction to avoid amplification of genomic DNA.

Primers were synthesized by Metabion International AG (Martinsried, Germany). Real-time RT-qPCR assays were performed in duplicate in 96-well plates. The thermal cycling conditions were 95 °C for 5 min for polymerase activation and the initial denaturation step, followed by 45 cycles with denaturation at 95 °C for 10s, annealing at 60 °C for 10s and extension at 72 °C for 10s. A melting curve analysis was recorded at the end of the amplification to evaluate the absence of contaminants or primer dimers. For each CC sample, gene expression levels of *HAS2*, *PTGS2*, *PTX3*, *TNFAIP6* and *GDF9* were calculated as fold-changes relative to *B2M* level calculated by 2^-ΔΔ*CT*^ method [46]. Depending on the transcript targeted, cumulus samples that did not reveal any expression data, either because of an amplification failure or because they did not show any expression of the five genes studied, were excluded from the analysis.

### Statistical analysis

NCSS (Number Cruncher Statistical System) 2007 (Kaysville, Utah, USA) program was used for statistical analysis. Data was reported as mean, standard deviation, standard error, frequency, percentage, minimum, maximum. Due to the paired nature of the data, generalized linear mixed models (GLMMs) were conducted.

First, to analyse if there was any correlation between follicular size, the potential of embryos to reach the blastocyst stage, blastocyst quality and gene expression levels, separate GLMMs were conducted. In these models, expression levels of genes were introduced as target variables, follicular size, the potential of embryos to reach blastocyst stage and blastocyst quality as fixed factors and subject id as a random factor.

Second, separate GLMMs with logit link were conducted to analyse the factors affecting embryos development into eRBs or into TQ or GQ blastocysts. In these models, eRB and blastocyst quality were introduced as target variables; age, BMI, AMH, follicular size and expression levels of genes as fixed factors and subject id as a random factor.

Third, separate GLMMs with logit link were conducted to analyse any correlation between gene expression levels and implantation. In these models, implantation was introduced as a target variable; age, BMI, AMH, follicular size and expression levels of genes as fixed factors and subject id as a random factor.

A *p* value of *p* < 0.05 was accepted as statistically significant.

## Results

### Follicular size and CC gene expression

When expression levels of genes were evaluated according to follicular size only, *PTGS2* was found to be significantly up-regulated in CCs deriving from large follicles when compared to small follicles (*p* < 0.001) (Table 1).

**Table 1:** CC gene expression levels regarding follicular size and embryo development on day5

Concentrations		*TNFAIP6*	*PTGS2*	*HAS2*	*PTX3*	*GDF9*
		Mean (se)	Mean (se)	Mean (se)	Mean (se)	Mean (se)
Follicular size	n	1195	1205	1212	1110	742
	Small	0.629 (0.564)	0.281 (0.027)	0.631 (0.287)	0.011 (0.004)	1.075 (0.297)
	n	1061	1066	1072	975	648
	Large	0.956 (0.784)	0.434 (0.047)	0.922 (0.350)	0.014 (0.003)	0.997 (0.255)
p^a^		0.204	< 0.001^**^	0.193	0.371	0.847
eRB	n	472	478	482	439	295
	Small	1.163 (1.065)	0.284 (0.027)	0.710 (0.339)	0.010 (0.003)	1.217 (0.533)
	n	605	609	612	559	370
	Large	0.790 (0.637)	0.399 (0.038)	0.811 (0.356)	0.013 (0.003)	0.966 (0.347)
p^a^		0.451	< 0.001^**^	0.036^*^	0.344	0.701
eFRB	n	723	727	730	671	447
	Small	0.235 (0.245)	0.270 (0.031)	0.538 (0.254)	0.013 (0.006)	0.987 (0.356)
	n	456	457	460	416	278
	Large	1.376 (1.193)	0.480 (0.082)	1.172 (0.562)	0.016 (0.005)	1.047 (0.383)
p^a^		0.274	0.010^*^	0.249	0.266	0.911
TQ or GQ Blastocysts	n	253	258	260	237	151
	Small	0.726 (0.506)	0.275 (0.031)	0.514 (0.127)	0.011 (0.003)	0.415 (0.323)
	n	349	353	354	325	212
	Large	0.727 (0.506)	0.388 (0.040)	0.562 (0.126)	0.013 (0.005)	0.410 (0.211)
p^a^		0.562	0.002^**^	0.060	0.714	0.956
Arrested embryos or BQ Blastocysts	n	942	947	952	873	591
	Small	0.684 (0.631)	0.274 (0.030)	0.649 (0.345)	0.011 (0.004)	1.243 (0.361)
	n	712	713	718	650	436
	Large	0.978 (0.841)	0.445 (0.059)	1.001 (0.414)	0.013 (0.003)	1.282 (0.369)
p^a^		0.217	0.003^**^	0.323	0.466	0.942

Studied genes are: tumour necrosis factor alpha induced protein 6 (*TNFAIP6*), prostaglandin-endoperoxide synthase 2 (*PTGS2*), hyaluronan synthase 2 (*HAS2*), pentraxin-related protein 3 (*PTX3*) and growth differentiation factor 9 (*GDF9*)

*eRB* embryos reaching blastocyst, *eFRB* embryos failed to reach blastocyst, *TQ* top quality blastocyst, *GQ* good quality blastocyst, *BQ* bad quality blastocyst

^**^p < 0.01; *p<0.05

^a^GLMM (Generalized linear mixed model) se: standard error

### CC gene expression, embryos reaching blastocyst stage and follicular size

Expression levels of the five genes were analysed in embryos that reached blastocyst stage (eRB) regarding follicular size. For embryos developing to blastocyst and originating from large follicles, *HAS2* and *PTGS2* expressions were significantly up-regulated in the associated CCs when compared to those originating from small follicles (*p* < 0.001; *p* = 0.036, respectively) (Table 1).

### CC gene expression, blastocyst quality and follicular size

For embryos becoming TQ or GQ blastocysts and originating from large follicles, *PTGS2* expression was up-regulated in the associated CCs when compared to small follicles (*p* = 0.002) (Table 1). However, a significant up-regulation for *PTGS2* was also observed in CCs of oocytes originating from large follicles and that developed to BQ blastocysts or arrested embryos, when compared to small follicles (*p* = 0.003) (Table 1). Again, a significant up-regulation for *PTGS2* was also observed for CCs of eFRB embryos originating from large follicles when compared to small follicles (*p* = 0.010) (Table 1).

### CC gene expressions evaluated with patient-specific variables regarding eRB or TQ + GQ blastocysts

Generalized Linear Mixed Models (GLMMs) with logit link were conducted to analyse the factors correlating with the development to an eRB and to TQ + GQ blastocysts. In these models, which were separately done for the five selected genes, age, BMI, AMH and follicular size were introduced as patient-specific variables and were simultaneously analysed with each CC gene expression.

For the model analyzing the factors affecting the development to an eRB, CC expression levels of *TNFAIP6*, *PTGS2*, *PTX3*, *HAS2* and *GDF9* were not found to be significant (Table 2). The only variable correlating with eRB for each of these five models was the follicular size (*p* < 0.001) (Table 2).

**Table 2:** CC expression of the studied genes regarding clinical variables and development into eRBs and TQ + GQs

		eRB		TQ + GQ	
		*p*	OR (95% CI)	*p*	OR (95% CI)
*TNFAIP6*	Age	0.790	0.996 (0.965, 1.028)	0.178	0.979 (0.948, 1.010)
	BMI	0.837	1.005 (0.957, 1.055)	0.324	1.025 (0.976, 1.075)
	AMH	0.195	1.045 (0.977, 1.118)	0.043^*^	1.083 (1.003, 1.169)
	^a^Follicle size (large)	< 0.001^**^	2.146 (1.760, 2.617)	< 0.001^**^	1.877 (1.512, 2.329)
	*TNFAIP6*	0.438	1.001 (0.997, 1.005)	0.081	0.988 (0.974, 1.002)
*PTGS2*	Age	0.817	0.996 (0.966, 1.027)	0.179	0.979 (0.950, 1.010)
	BMI	0.976	1.001 (0.953, 1.051)	0.435	1.019 (0.971, 1.070)
	AMH	0.214	1.043 (0.975, 1.116)	0.060	1.076 (0.997, 1.160)
	^a^Follicle size (large)	< 0.001^**^	2.147 (1.760, 2.618)	< 0.001^**^	1.863 (1.503, 2.308)
	*PTGS2*	0.439	0.966 (0.887, 1.054)	0.670	0.978 (0.881, 1.085)
*PTX3*	Age	0.873	0.997 (0.966, 1.029)	0.084	0.973 (0.943, 1.004)
	BMI	0.831	0.995 (0.948, 1.044)	0.492	1.017 (0.969, 1.068)
	AMH	0.149	1.051 (0.982, 1.126)	0.026^*^	1.089 (1.010, 1.174)
	^a^Follicle size (large)	< 0.001^**^	2.162 (1.751, 2.671)	< 0.001^**^	1.880 (1.506, 2.346)
	*PTX3*	0.720	0.698 (0.097, 4.998)	0.847	0.831 (0.126, 5.446)
*HAS2*	Age	0.831	0.997 (0.967, 1.028)	0.147	0.978 (0.949, 1.008)
	BMI	0.982	1.001 (0.954, 1.050)	0.401	1.021 (0.973, 1.070)
	AMH	0.163	1.048 (0.981, 1.120)	0.037^*^	1.081 (1.005, 1.163)
	^a^Follicle size (large)	< 0.001^**^	2.120 (1.741, 2.582)	< 0.001^**^	1.859 (1.504, 2.297)
	*HAS2*	0.511	0.995 (0.982, 1.009)	0.043^*^	0.955 (0.913, 0.998)
*GDF9*	Age	0.785	0.995 (0.960, 1.031)	0.208	0.976 (0.939, 1.014)
	BMI	0.715	0.990 (0.937, 1.045)	0.348	1.027 (0.971, 1.085)
	AMH	0.103	1.078 (0.985, 1.180)	0.067	1.088 (0.994, 1.190)
	^a^Follicle size (large)	< 0.001^**^	2.145 (1.683, 2.734)	< 0.001^**^	1.913 (1.461, 2.505)
	*GDF9*	0.912	1.001 (0.987, 1.015)	0.049^*^	0.975 (0.952, 0.999)

(Studied genes are: tumour necrosis factor alpha induced protein 6 (*TNFAIP6*), prostaglandin-endoperoxide synthase 2 (*PTGS2*), hyaluronan synthase 2 (*HAS2*), pentraxin-related protein 3 (*PTX3*) and growth differentiation factor 9 (*GDF9*)). Embryos reaching blastocyst stage (eRB); top (TQ) or good quality (GQ) blastocysts

^*^p < 0.05; ^**^p < 0.01

^a^Small follicular size was selected as a reference category

Generalized Linear Mixed Models (GLMMs) with logit link were also conducted to analyse the factors correlating with blastocyst quality (TQ + GQ). Follicular size was found to be significantly correlated with TQ + GQ in all of these five models (*p* < 0.001), whereas AMH was found to be a variable correlated with the potential of embryos becoming TQ + GQ blastocysts in only three models out of five (*TNFAIP6* (*p* = 0.043); *PTX3* (*p* = 0.026) and *HAS2* (*p* = 0.037)) (Table 2). Blastocyst quality was significantly associated with CC gene expression of *HAS2* and *GDF9* (p = 0.043; *p* = 0.049, respectively) but not with that of *TNFAIP6*, *PTGS2* and *PTX3* (Table 2).

### Follicle size, CC gene expression and implantation

Before starting our two-part study, we conducted a pilot study in order to calculate the minimum number of fertilized oocytes which would be sufficient to detect a significant difference between follicle size and blastulation rates. The results revealed that top and good quality blastocyst rates for large and small follicles were 45 and 32%, respectively. A power analysis indicated that, for an alpha level of 0.05 and a beta level of 0.20 (power = 0.80), 438 fertilized oocytes were sufficient to detect a significant difference between follicle size groups in terms of blastulation rate [1]*.* But, in spite of the large cohort, the number of resultant blastocysts with known implantation data would probably not be sufficient to detect a significant correlation between follicular size, gene expression and implantation. However, recognizing that implantation rates are of interest, further separate GLMMs with logit link were conducted to analyse any correlation between gene expression levels, patient-specific variables (age, BMI, AMH and follicular size) and implantation. Results from these models indicated that neither patient-specific variables nor the five studied CC gene expressions had a statistically significant correlation with implantation (*p* > 0.05). This supports our previous findings, published in an article of 2017 [1]*,* where we discussed the results of the first part of this study into the morphokinetic development of embryos in relation to follicle diameter and homogeneity of follicular development. Although oocytes developing from large follicles were more than twice as likely to develop into an eRB than oocytes from small follicles, once the blastocyst stage was achieved, implantation rates were not significantly different between embryos coming from large or small follicles (Additional file 1: Table S3).

## Discussion

Morphological criteria which are currently used to describe intra- and extra-cytoplasmic features of human oocytes are not sufficient to accurately and non-invasively predict developmentally competent oocytes [47]. Defined gene expression signatures of oocyte competence acquired during maturation by the surrounding somatic cells would be valuable in predicting the developmental potential of oocytes. The developmental competence of oocytes is indeed acquired progressively during folliculogenesis and is well-known to rely on the bidirectional communication between oocyte and CCs, which deliver nutrients and regulatory molecular signals to promote both oocyte nuclear and cytoplasmic maturation. Our study investigates if there is any association between putative oocyte competence markers in CCs and the developmental competence of oocytes as evaluated by the quality of blastocysts.

Cumulus expansion in vivo is induced by the preovulatory LH surge and requires expression of transcripts encoding hyaluronan synthase 2 (*HAS2*), prostaglandin-endoperoxide synthase 2 (*PTGS2*), pentraxin-related protein 3 (*PTX3*) and tumour necrosis factor alpha induced protein 6 (*TNFAIP6*) [48]. During maturation, oocyte secretory factors (*GDF9* and *BMP15*) act upon CCs and up-regulate mRNA expressions of the above-mentioned cumulus expansion enabling factors [49].

Nivet and colleagues [24], evaluated the relationship between the embryonic outcome of oocytes, follicle volume and transcriptomic signature and concluded that transcriptional characteristics of follicles varied according to their size and that size should be considered to better discriminate between follicles containing oocytes with high developmental competence. In the present study reporting a series of 2495 COCs punctured from 184 young and good prognosis patients, follicular size significantly correlated with the potential of an oocyte to develop into blastocyst: oocytes developing from large follicles were more than twice as likely to develop into an eRB than oocytes from small follicles (*p* < 0.001).

When expression levels of genes were evaluated according to follicular size only, *PTGS2* was significantly up-regulated in large follicles whereas no such significant difference in gene expression was observed in *TNFAIP6, HAS2, PTX3* and *GDF9* (Table 1, Follicular size). Again, when evaluating gene expression levels while taking follicular size into account, significant differences were found only in *PTGS2* and *HAS2* in embryos originating from large follicles and reaching to blastocyst stage (Table 1, eRB). Only *PTGS2* was significantly up-regulated in top or good quality blastocysts originating from oocytes coming from large follicles (Table 1, TQ or GQ Blastocysts). However, *PTGS2* was also upregulated in embryos failing to reach blastocyst, bad quality blastocysts and arrested embryos (Table 1).

Thus, *PTGS2* expression in CCs correlated significantly with follicular size being up regulated in large follicles regardless of whether embryos arrested or were slow growing or whether blastocyst stage was reached or not and whether blastocysts were poor, good or top quality. These findings seem to indicate that differences of gene expression of *PTGS2* were related to follicular size rather than to embryo quality. This demonstrates that up-regulation of *PTGS2* in large follicles does not necessarily indicate oocyte competence.

In a study by Ekart and colleagues [19], in which a group of 25 women below the age of 38 underwent an rFSH-stimulated ICSI treatment, a total of 270 COCs were analysed. The authors found that 99.7% of COC retrieved within each woman involved in their study were significantly different from each other regarding CC mRNA levels of eight candidate genes (*HAS2, FSHR, SLC2A4, ALCAM, SFRP2, VCAN, NRP1* and *PR*) and cell composition [19]. The authors associate this heterogeneity either with an artefact during the mechanical isolation and enzymatic dissection process or to variations in granulosa cell numbers in developing follicles. Our study may indicate that this heterogeneity could also be related to size discrepancies within a follicular cohort stimulated exogenously by gonadotropins.

Two genes, *HAS2* and *GDF9*, showed significantly higher expression in CCs from oocytes that progressed to TQ or GQ blastocysts. Our results support a relationship between *HAS2* and *GDF9* expression in CCs and the developmental competence of oocytes. According to the GLMMs with logit link, one unit increase in *HAS2* decreased the probability of obtaining a TQ + GQ blastocyst by 8.7%. Similarly, one unit increase in *GDF9* decreased the probability of obtaining a TQ + GQ blastocyst by 2.5%. No such correlation was found in other gene expressions studied. However, once again this correlation was seen only when the genes were evaluated together with follicular size and the patient specific variables of age, BMI and AMH. Although gene expression of *HAS2* and *GDF9* correlated with blastocyst quality, the only variable correlating with eRB and TQ and GQ blastocysts for each of the five GLMM models was follicular size. Furthermore, the correlation was stronger. These findings support the clinical implications of the first part of our study [1] that follicle size is a strong indicator of the likelihood of an embryo proceeding to blastocyst.

As the nuclear and cytoplasmic competence are acquired during follicular growth, which requires the bidirectional cross-talk between the oocyte itself and CCs, CC gene expression of essential genes can be expected to be altered. Thus, it can be speculated that follicular size is fundamental when analysing CC expression data and that markers of oocyte competence based on CC studies have to include follicular size to be rigorously accurate.

The complexity related to modifiers of gene expression is illustrated in the recent literature [32]. In a randomized study, Cruz and colleagues (2017) showed that the type of gonadotropin used during controlled ovarian stimulation (follicle-stimulating hormone (FSH), urinary FSH, or human menopausal gonadotropin (hMG)) induce differential gene expression in human CCs [50]. Moreover, *PTGS2* was shown to be down-regulated in CCs of infertile women with endometriosis [51]. Patient age affects differential mRNA gene expression in CCs [52–54]. Patient BMI is also a modifier of CC gene expression in both polycystic ovarian syndrome (PCOS) [44] and non-PCOS women [54]. CC gene expression from different patients were studied, and it is known that factors such as different stimulation protocols, patient characteristics and transfer techniques may impact either CC gene expression or implantation [44, 50–56].

Therefore, in our study, patients were selected with inclusion criteria as similar as was possible in a clinical context. Although some minor variations (e.g. BMI) were unavoidable, bias in the participants selection was minimized by including only antagonist cycles in the study, by administering GnRH antagonist when the size of the follicle was at least 12 mm, but never exceeding 13 mm. Although there was some variation in the FSH dose (150–225 IU) depending on BMI, cases with more extreme dosages were not included. Furthermore, PCOS patients with an abnormally high number of COCs were excluded [44], because abnormal gene expression profiles in human ovaries from polycystic ovary syndrome patients have been described in the literature [57]. Finally, severe male infertility cases, where sperm might have played a critical role, were excluded.

Thus, this study was conducted using a homogeneous group of young and good prognosis patients and generalized linear mixed models (GLMMs) were applied in order to correct the expression data of each oocyte regarding the clinical characteristics of each patient included in the study. Hence, unlike a more basic statistical evaluation in which each oocyte is considered to be independent, this paired analysis corrected for the follicular cohort effect, giving more reliable results.

A correlation was found in this study between blastocyst quality and CC gene expressions of *HAS2* and *GDF9* by GLMM analyses. Increased levels of *GDF9* and *BMP15* expression have both been associated with positive pregnancy as well as with mature oocytes, fertilization rate and embryo quality [43]. Analyzing *GDF9* and *BMP15* expression by qRT-PCR, Li and colleagues [32] utilized one of the largest samples sizes to date, using 2426 CC masses from 196 patients. Furthermore, *HAS2* is considered to be a human pregnancy or live birth candidate biomarker with conflicting differential mRNA expression by qRT-PCR [2, 19, 31, 32].

Finally, to answer the questions asked when beginning the study; although gene expression of *HAS2* and *GDF9* correlated with blastocyst quality when separately evaluated with follicular size and the patient specific variables of age, BMI and AMH, the only variable correlating with eRB and TQ and GQ blastocysts for each of these five models was follicular size. Furthermore, the correlation was stronger.

## Conclusions

In conclusion, follicular size correlated significantly with blastocyst quality and viability. Embryos coming from large follicles were almost twice as likely as those coming from small follicles to develop into TQ + GQ blastocysts suitable for transfer or freezing. There was also a correlation between blastocyst quality and CC gene expressions of *HAS2* and *GDF9*, but only when evaluated together with follicular size, age, BMI and AMH, no such correlation was found in the case of other gene expressions studied. The only variable correlating with eRB and TQ and GQ blastocysts for each of the five models including the expression levels of the five genes studied was follicular size. Our findings suggest that differences in gene expression could be related to follicular size rather than to embryo quality.

## Additional file

Additional file 1 **Table S1.** Patients’ characteristics and outcomes. **Table S2.** Oligonucleotide primer sequences used for real-time PCR in this study. **Table S3.** Separate analyses of CC expression of the studied genes regarding clinical variables and implantation. (DOCX 20 kb)

## Abbreviations

BMI: Body Mass Index
BQ: Bad Quality blastocyst
CCs: Cumulus Cells
COC: Cumulus Oocyte Complex
eFRB: embryos Failed to Reach Blastocyst
eRB: embryos Reaching Blastocyst
*GDF9*: Growth Differentiation Factor 9
GLMMs: Generalized linear mixed models
GQ: Good quality blastocyst
*HAS2*: Hyaluronan Synthase 2
MII: Metaphase II oocyte
OPU: Oocyte Pick-Up
*PTGS2*: Prostaglandin-Endoperoxide Synthase 2
*PTX3*: Pentraxin-related protein 3
*TNFAIP6*: Tumour Necrosis Factor Alpha Induced Protein 6
TQ: Top quality blastocyst
