# Supplementary material for: Is there a correlation between follicle size and gene expression in cumulus cells and is gene expression an indicator of embryo development?
Source: Reprod Biol Endocrinol. 2018 Jul 21;16:69. doi: 10.1186/s12958-018-0388-0 (PMC6054838; doi:10.1186/s12958-018-0388-0)
Supplement: Supplementary file 1 — Table S1. Patients’ characteristics and outcomes. Table S2. Oligonucleotide primer sequences used for real-time PCR in this study. Table S3. Separate analyses of CC expression of the studied genes regarding clinical variables and implantation. (DOCX 20 kb) [file 12958_2018_388_MOESM1_ESM.docx]

Additional File 1**: Table S1** Patients’ characteristics and outcomes.

| **Age (years)** | Min-Max (Median) | 18-38 (31) |
| --- | --- | --- |
|  | Mean±SD | 30.81±4.26 |
| **AMH (ng/ml)** | Min-Max (Median) | 0.17-8.6 (2.80) |
|  | Mean±SD | 3.15±1.80 |
| **BMI (kg/m^2^)** | Min-Max (Median) | 16.3-29.7 (23.3) |
|  | Mean±SD | 23.68±3.00 |
| **+bhCG; n (%)** |  | 122 (66.3) |
| **Ongoing pregnancy; n (%)** |  | 98 (53.3) |
| **Live birth; n (%)** |  | 94 (51.1) |

Additional file 1**: Table S2** Oligonucleotide primer sequences used for real-time PCR in this study.

| **Gene Name** | **GenBank Accession Number** | **Primer Sequence (5’ to 3’)** |
| --- | --- | --- |
| ***TBP*** | NM_001172085.1 | *F: AATCCCAAGCGGTTTGCTG  **R: AGCATATTTTCTTGCTGCCAGTCT |
| ***B2M*** | NM_005254549.3 | F: CTCGCTCCGTGGCCTTAG  R: ATGGATGAAACCCAGACACATAGC |
| ***GDF9*** | NM_005260.5 | F: TCCAATAGAAGTCACCTCTACAACAC  R: TGCTAGAAGACTTTGGCTCCT |
| ***PTX3*** | NM_002852.3 | F: CAGTGCTCTGGACGAGCT  R: TCTTGGAACGCATTGGGA |
| ***HAS2*** | NM_005328 | F: GGCATCCAGCACTGGACAAA  R: ATTCAGGCCACAGAACAAAACC |
| ***PTGS2*** | NM_000963.3 | F: GTGTCAAAGGTAAAAAGCAGCTTC  R: CCGTAGATGCTCAGGGACT |
| ***TNFAIP6*** | NM_007115.3 | F: CATATGGCTTGAACGAGCAG  R: ATCCATCCAGCAGCACAG |

*Forward **Reverse

(Gene names are: tumour necrosis factor alpha induced protein 6 (*TNFAIP6*), prostaglandin-endoperoxide synthase 2 (*PTGS2*), hyaluronan synthase 2 (*HAS2*), pentraxin-related protein 3 (*PTX3*), growth differentiation factor 9 (*GDF9*), β2 microglobulin (*B2M*) and TATA-binding protein (*TBP*)).

Additional file 3**: Table S3** Separate analyses of CC expression of the studied genes regarding clinical variables and implantation.

|  | **Implantation** | |
| --- | --- | --- |
|  | **p** | **OR (95% CI)** |
| **Age** | 0.159 | 0.931 (0.841, 1.029) |
| **BMI** | 0.980 | 1.002 (0.876, 1.146) |
| **AMH** | 0.570 | 0.932 (0.73, 1.191) |
| **‡Follicle size (large)** | 0.500 | 1.24 (0.661, 2.325) |
| ***TNFAIP6*** | 0.894 | 16.553 (0.001, 2.019*10^19^) |
| **Age** | 0.138 | 0.928 (0.84, 1.025) |
| **BMI** | 0.806 | 1.017 (0.887, 1.166) |
| **AMH** | 0.616 | 0.94 (0.735, 1.201) |
| **‡Follicle size (large)** | 0.540 | 1.213 (0.651, 2.263) |
| ***PTGS2*** | 0.297 | 1.675 (0.631, 4.449) |
| **Age** | 0.100 | 0.922 (0.837, 1.016) |
| **BMI** | 0.800 | 1.017 (0.891, 1.161) |
| **AMH** | 0.407 | 0.908 (0.721, 1.143) |
| **‡Follicle size (large)** | 0.389 | 1.31 (0.706, 2.431) |
| ***HAS2*** | 0.179 | 1.238 (0.904, 1.696) |
| **Age** | 0.088 | 0.916 (0.828, 1.013) |
| **BMI** | 0.611 | 1.037 (0.901, 1.193) |
| **AMH** | 0.385 | 0.9 (0.707, 1.144) |
| **‡Follicle size (large)** | 0.105 | 1.779 (0.884, 3.577) |
| ***PTX3*** | *0.076* | 0.001 (0, 2.224) |
| **Age** | 0.199 | 0.928 (0.827, 1.041) |
| **BMI** | 0.760 | 1.025 (0.874, 1.201) |
| **AMH** | 0.866 | 0.974 (0.713, 1.33) |
| **‡Follicle size (large)** | 0.521 | 1.316 (0.563, 3.075) |
| ***GDF9*** | 0.635 | 0.968 (0.844, 1.11) |

^‡^Small follicular size was selected as a reference category.

(Studied genes are: tumour necrosis factor alpha induced protein 6 (*TNFAIP6*), prostaglandin-endoperoxide synthase 2 (*PTGS2*), hyaluronan synthase 2 (*HAS2*), pentraxin-related protein 3 (*PTX3*) and growth differentiation factor 9 (*GDF9*)).
